# Dexamethasone induced miR-155 up-regulation in differentiating 3T3-L1 preadipocytes does not affect adipogenesis

**DOI:** 10.1038/s41598-018-19704-4

**Published:** 2018-01-19

**Authors:** Vian Peshdary, Ella Atlas

**Affiliations:** 1 Environmental Health Science and Research Bureau, Health Canada, 50 Colombine Driveway, Ottawa, Ontario, Canada; 2 Department of Biochemistry, Microbiology, and Immunology, University of Ottawa, Ottawa, Ontario, Canada

## Abstract

Dexamethasone is a synthetic glucocorticoid that is widely used as an adipogenic inducer in both murine and human *in vitro* models. Glucocorticoids have been shown to regulate early transcriptional events in adipogenesis. MicroRNAs (miRNAs) have been also implicated in the regulation of preadipocyte differentiation; however, the effects of glucocorticoids on miRNA expression levels during this process have not been studied. In this study we investigated the effects of glucocorticoids on the expression levels of miR-155 in differentiating 3T3-L1 preadipocytes. We found that miR-155 levels were up-regulated (2.4-fold) by glucocorticoids in differentiating 3T3-L1 preadipocytes, and this enhancement was abolished in the presence of RU486, a glucocorticoid receptor antagonist. In contrast, treatment with rosiglitazone, another adipogenic inducer decreased the expression levels of miR-155 in these cells. Further, our data show that endogenous miR-155 is unlikely to be involved in adipogenesis as we show that both dexamethasone and rosiglitazone induced adipogenesis to similar levels. Furthermore, using miR-155 inhibitor, we showed that the dexamethasone mediated miR-155 enhancement did not alter adipogenesis. Our data show that dexamethasone but not rosiglitazone increases miR-155 expression and that the increased expression of miR-155 is not involved in the dexamethasone-mediated adipogenesis in the 3T3-L1 model.

## Introduction

Glucocorticoids are a class of corticosteroids that can promote adipogenesis *in vivo*. Increased levels of the glucocorticoid steroid hormone cortisol promotes visceral adipose tissue expansion in humans^1^. Further, patients with Cushing’s syndrome, who exhibit sustained high cortisol levels, develop visceral obesity and metabolic syndrome^2^. Similarly, exogenous glucocorticoid therapy leads to the development of truncal obesity^3^.

The effects of glucocorticoids are mediated by activation of the glucocorticoid receptor (GR) - a nuclear hormone receptor of the steroid receptor subfamily^4^. GR can positively and negatively regulate the expression of genes involved in cell differentiation, inflammation, lipid metabolism, glucose uptake, and others^5^. Specifically, GR is a transcription factor involved in preadipocyte differentiation of cells from murine and human origins^6,7^. The early transcriptional events mediated by GR in preadipocyte differentiation have been previously elucidated^8^. However, to the best of our knowledge, GR effects on modulating expression levels of microRNAs (miRNAs) that contribute to adipogenesis are not known. MiRNAs are small non-coding RNAs that regulate gene expression in different biological processes such as apoptosis, development, and cell proliferation/differentiation^9^. They regulate gene expression by supressing mRNA transcripts of protein coding genes and/or by inhibiting protein translation^10^.

In animal models of obesity, miR-155 has been shown to be involved in promoting inflammation and insulin resistance^11^. In addition, in leptin deficient and diet induced obese mice levels of miR-155 were increased in visceral fat pad (epididymal), suggesting that miR-155 may have a role in adipogenesis^12^. Furthermore, it has been shown that levels of miR-155 were increased both in preadipocytes and adipocytes when cells were treated with tumor necrosis factor (TNF) α^13,14^. One of these studies suggested that TNF- α mediated increase in endogenous miR-155 supressed 3T3-L1 adipogenesis^13^. The same study suggested that ectopic expression of miR-155 inhibited mouse 3T3-L1 preadipocytes. Another study using human mesenchymal stromal cells (hMSCs) showed that over-expression of miR-155 reduced the extent of differentiation into adipocytes^13,15^. As such, the role of miR-155 in adipogenesis has been mainly studied either under inflammatory conditions or by ectopic over-expression. In this study we evaluated the role of the dexamethasone-induced endogenous miR-155 in adipogenesis and attempted to elucidate the role of this increase in the differentiation process mediated by glucocorticoids.

## Results

### Dexamethasone, but not rosiglitazone, induces miR-155 expression in differentiating preadipocytes

**Figure 1:**
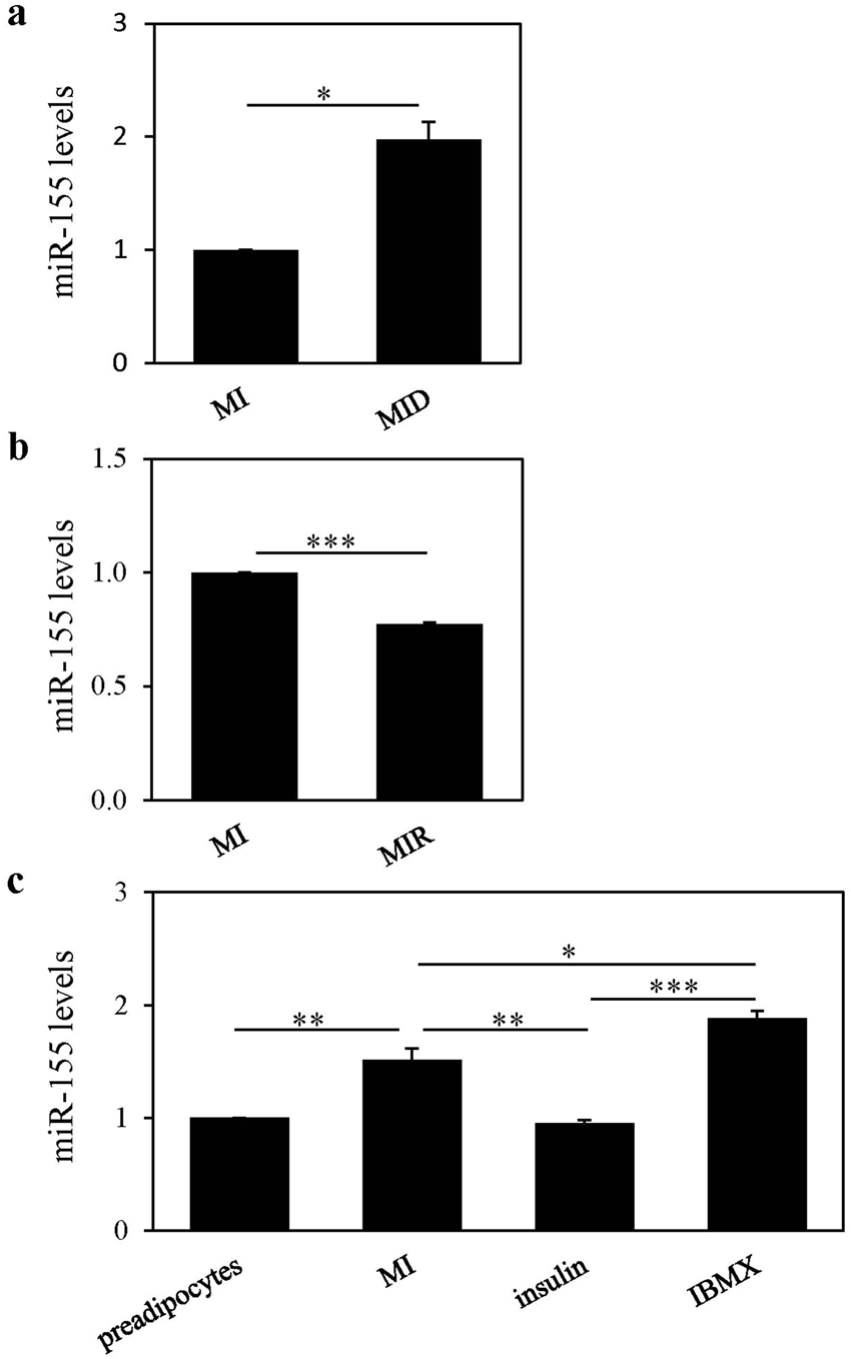
miR-155 expression was induced by dexamethasone, but not rosiglitazone, in differentiating preadipocytes. (**a**) Murine 3T3-L1 preadipocytes were induced to differentiate in the presence of 500 µM IBMX (M), 100 nM insulin (I) and supplemented with either solvent control (MI) or 250 nM dexamethasone (MID). (**b**) Murine 3T3-L1 preadipocytes were induced to differentiate in the presence of 500 µM IBMX (M), 100 nM insulin (I) and supplemented with either solvent control (MI) or 5 μM rosiglitazone (MIR). (**c**) Murine 3T3-L1 preadipocytes were treated with 500 µM IBMX (M) and/or 100 nM insulin (I). Otherwise, preadipocytes were left in media, not including differentiation inducers (preadipocytes). After 24 hours, miRNA was isolated, and the expression levels of miR-155 were quantified by real-time qPCR. Levels were normalized to endogenous RNU6 and expressed as fold over MI. Results from 3 separate experiments are graphically represented as mean ± S.E.M. *Denotes p < 0.05, **denotes p < 0.01, and ***denotes p < 0.001, as assessed by paired t-test (**a** and **b**) or one-way ANOVA with Tukey’s post-hoc tests (**c**).

We have previously performed a miRNA array experiment to evaluate the specific effects of glucocorticoids on miRNA profiles in differentiating 3T3-L1 preadipocytes that were induced to differentiate for 24 hours in MI (IBMX and insulin) compared to MID (IBMX, insulin, and dexamethasone) conditions. The miRNA screen resulted in a number of false positive changes in miRNA expression in response to dexamethasone as confirmed by real time-qPCR (Supplementary Table S1 and Fig. S1) and only miR-155 was confirmed. Indeed, dexamethasone (MID) significantly increased miR-155 levels by 2-fold compared to the basal, MI condition after 24 hours (Fig. 1a). To understand whether this dexamethasone-mediated miR-155 induction was part of the adipogenic process we also assessed the effects of another adipogenic inducer, rosiglitazone, a peroxisome proliferator-activated receptor (PPAR) γ agonist^16^, on miR-155 levels during induction of differentiation. The standard protocol for differentiation of 3T3-L1 cell includes insulin, 3-Isobutyl-1-methylxanthine (IBMX), and dexamethasone^16^. However, we have previously shown that the addition of a PPAR γ agonist to this differentiation cocktail in place of dexamethasone can also promote lipid accumulation to the same extent^16^. Interestingly, we found that rosiglitazone (MIR; IBMX, insulin and rosiglitazone) did not increase, but significantly decreased the levels of miR-155 by 23% compared to basal MI control after 24 hours treatment (Fig. 1b). Of note, we found that IBMX and Insulin (MI) significantly increased miR-155 levels by 1.5-fold compared to preadipocyte cultures that were not exposed to any differentiation inducers (Fig. 1c). Further, the MI-mediated miR-155 increase was attributed to the IBMX and not insulin (Fig. 1c).

**Figure 2:**
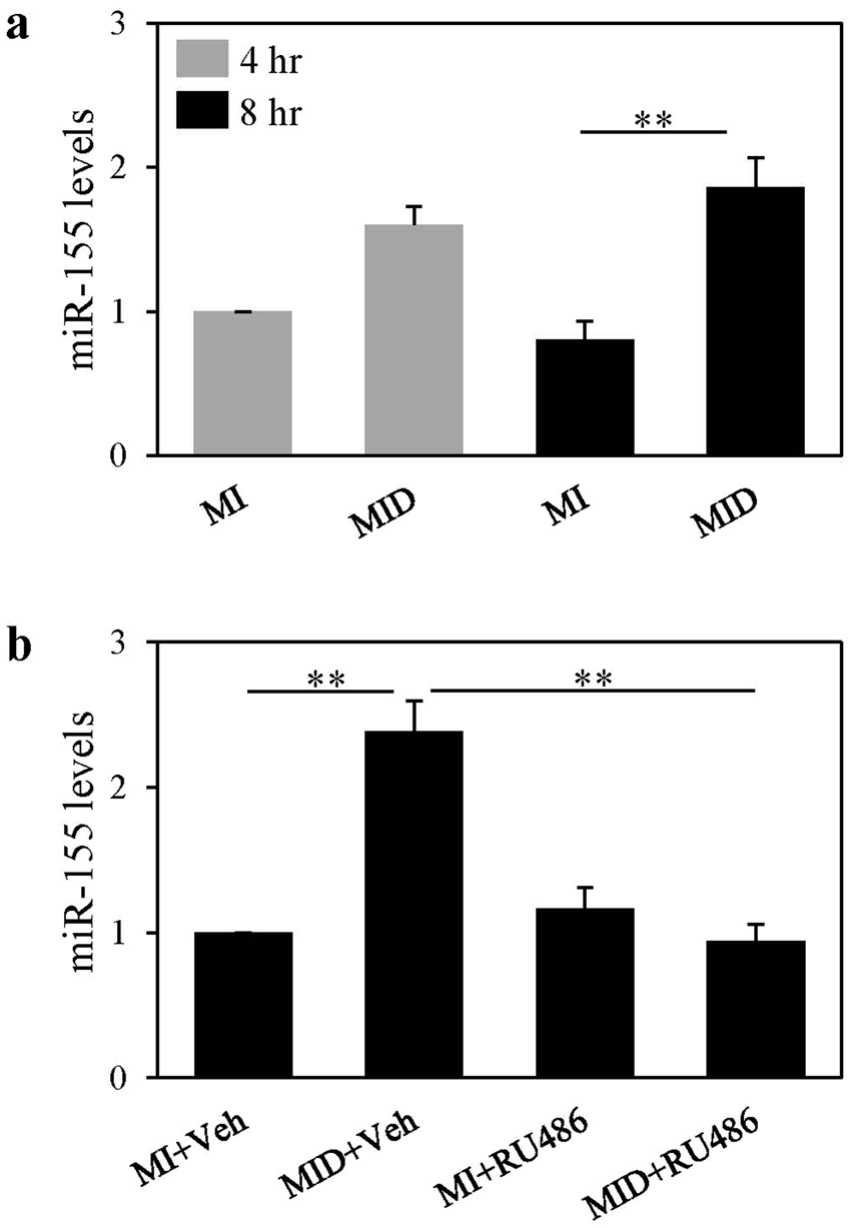
miR-155 is downstream of the glucocorticoid receptor in differentiating preadipocytes. (**a**) Murine 3T3-L1 preadipocytes were induced to differentiate in the presence of 500 µM IBMX (M), 100 nM insulin (I), and supplemented with either solvent control (MI) or 250 nM dexamethasone (MID) for 4 and 8 hours. At each time point, miRNA was isolated, and the expression levels of miR-155 were quantified by real-time qPCR. Levels were normalized to endogenous RNU6 and expressed as fold over MI at 4 hours. Results from 3 separate experiments are graphically represented as mean ± S.E.M. (**b**) Murine 3T3-L1 preadipocytes were induced to differentiate in the presence of 500 µM IBMX (M), 100 nM insulin (I), and supplemented with either solvent control (MI) or 250 nM dexamethasone (MID). Glucocorticoid receptor inhibitor RU486 (1 µM) or vehicle (Veh; ethanol) were added to MI and MID conditions as indicated. After 8 hours miRNA was isolated and, real-time qPCR quantified the expression levels of miR-155. Levels were normalized to endogenous RNU6 and expressed as fold over MI+Veh. Results from 3 separate experiments are graphically represented as mean ± S.E.M. **Denotes p < 0.01, as assessed by one-way ANOVA with Tukey’s post-hoc tests.

To further elucidate the dexamethasone-mediated temporal expression of miR-155 induction in differentiating preadipocytes we measured miR-155 levels at 4 and 8-hours post treatment. We found that miR-155 levels trended to increase at 4 hours, by 1.6-fold, and further increased to 2.4-fold by 8 hours of treatment with dexamethasone compared to basal controls at each time point (Fig. 2a).

### Dexamethasone mediated miR-155 induction is inhibited by a GR antagonist in differentiating preadipocytes

Next, we investigated whether GR activation is required for dexamethasone mediated miR-155 increased expression in differentiating preadipocytes. To address this, 3T3-L1 preadipocytes were induced to differentiate in MID versus MI conditions with or without the GR antagonist RU486^17^, for 8 hours. We showed once more that after 8 hours miR-155 levels were increased (2.4-fold) by dexamethasone compared to basal control and this increase was abolished in RU486 treated cultures (Fig. 2b). Taken together, these results suggest that miR-155 upregulation is mediated through GR transactivation in differentiating 3T3-L1 preadipocytes.

### Dexamethasone-mediated endogenous miR-155 up-regulation does not affect adipogenesis

**Figure 3:**
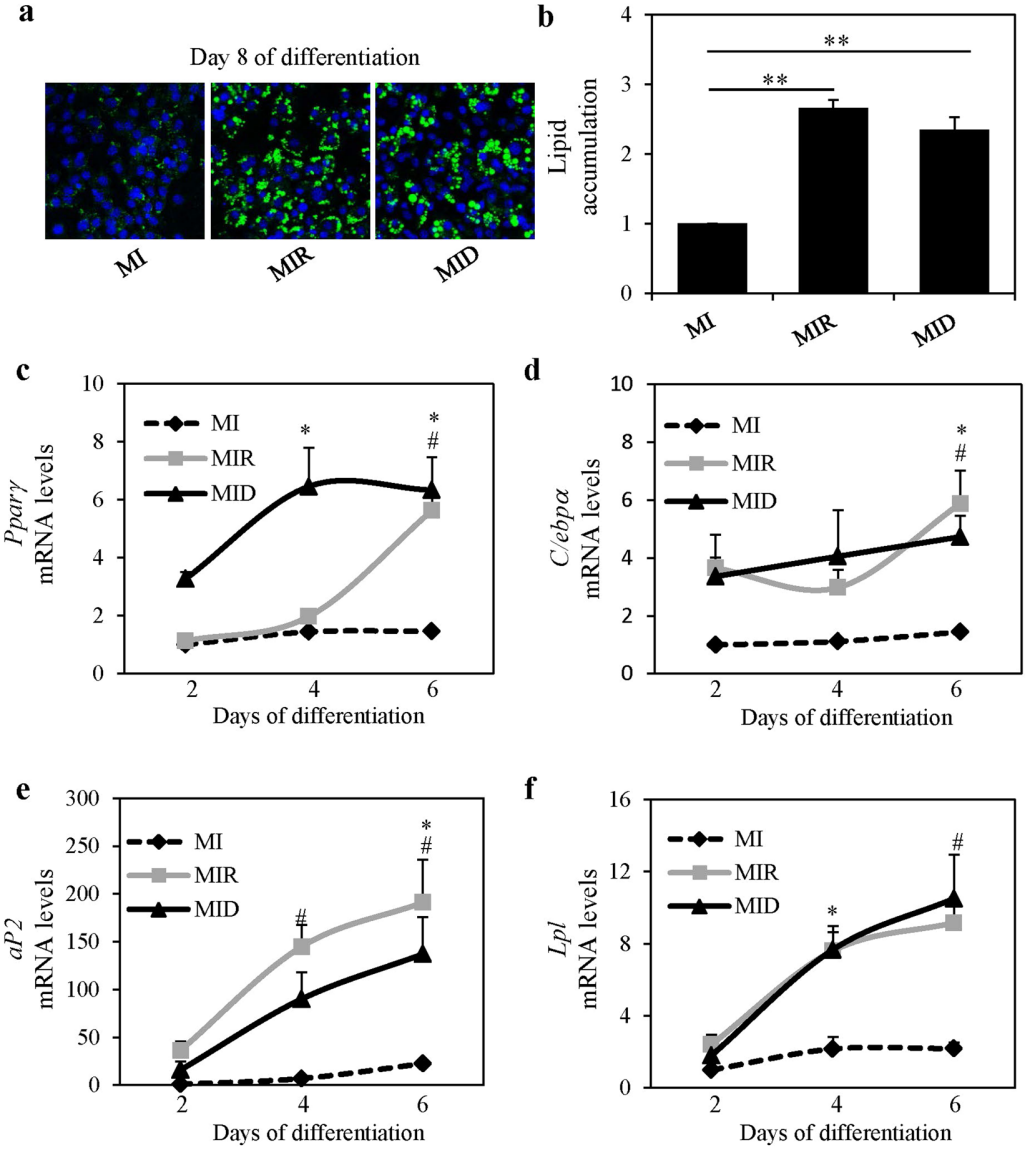
Dexamethasone and rosiglitazone induce adipogenesis to similar levels. Murine 3T3-L1 preadipocytes were induced to differentiate in the presence of 500 µM IBMX (M), 100 nM insulin (I), and supplemented with either solvent control (MI), 5 µM rosiglitazone (MIR), or 250 nM dexamethasone (MID). At day 8 of differentiation lipid accumulation was visualized using Nile Red (green) staining (**a**) and then quantified (**b**). Lipid accumulation was normalized to DAPI (blue) staining and expressed as fold over MI conditions. Results from 3 separate experiments are graphically represented as mean ± S.E.M. **Denotes p < 0.01 for indicated comparisons (**b**). At days 2, 4, and 6 of differentiation RNA was isolated, and the mRNA levels of and adipogenic markers, *Pparγ* (**c**), *C/ebpα* (**d**), *aP2* (**e**) and *Lpl* (**f**) were quantified by real-time qPCR. Levels were normalized to endogenous *β-actin* levels and expressed as fold over MI at day 2. Results from 4 separate experiments are graphically represented as mean ± S.E.M. *Denotes p < 0.05 when comparing MID conditions to MI at day 2 control and ^#^Denotes p < 0.05 when comparing MIR conditions to MI at day 2 control. Statistical analysis was performed by one-way ANOVA with Tukey’s post-hoc tests.

A previous study reported that exogenous overexpression of miR-155 in 3T3-L1 preadipocytes inhibited adipogenesis of the 3T3-L1 cells^13^. This group also reported that TNF-α mediated endogenous miR-155 induction in 3T3-L1 preadipocytes led to inhibition of adipogenesis^13^. Dexamethasone is a widely used adipogenic inducer both in murine and human cultures; therefore, we asked whether dexamethasone-mediated endogenous upregulation of miR-155 has a role in adipogenesis. Since we demonstrated that miR-155 levels increased with dexamethasone, but they were decreased by rosiglitazone in differentiating preadipocytes (Fig. 1a and b), we asked whether there was a difference in the adipogenic potential of dexamethasone versus rosiglitazone in this model system. We have previously shown that in 3T3-L1 preadipocytes induced to differentiate with MIR versus MID, there was no difference in lipid accumulation in the resulting adipocytes^16^. Here we show again that lipid accumulation was not changed in dexamethasone versus rosiglitazone treated cultures after 8 days of differentiations (Fig. 3a and b). In the present study, we also assessed the temporal mRNA levels of adipogenic transcription factors and adipogenic markers in 3T3-L1 preadipocytes differentiating in MIR versus MID conditions by real time-qPCR. We found that the mRNA levels of the transcription factors, *Pparγ* and *C/ebpα* increased with differentiation in both conditions compared to basal MI conditions (Fig. 3c and d). By the last day of differentiation *Pparγ* and *C/ebpα* mRNA expressions reached similar levels in both rosiglitazone and dexamethasone-treated cultures (Fig. 3c and d). It should be noted however, that dexamethasone mediated *Pparγ* mRNA increase occurred earlier in differentiation compared to the rosiglitazone treated cells (Fig. 3c). Further, the mRNA levels of adipogenic markers *fatty acid binding protein 4* also known as *aP2* and *lipoprotein lipase* (*Lpl*) increased with differentiation in both conditions (MIR versus MID) relative to basal control. Also, these increases occurred at comparable levels in rosiglitazone- versus dexamethasone- treated cultures (Fig. 3e and f). The levels of *aP2* trended to a higher increase in rosiglitazone- versus dexamethasone- treated cultures (Fig. 3e). Taken together, these results suggest that dexamethasone-mediated miR-155 induction may not be related to adipogenesis since both rosiglitazone and dexamethasone treatments resulted in the same levels of differentiation, even though miR-155 was increased only by dexamethasone and was even decreased by rosiglitazone (Fig. 1a and b).

**Figure 4:**
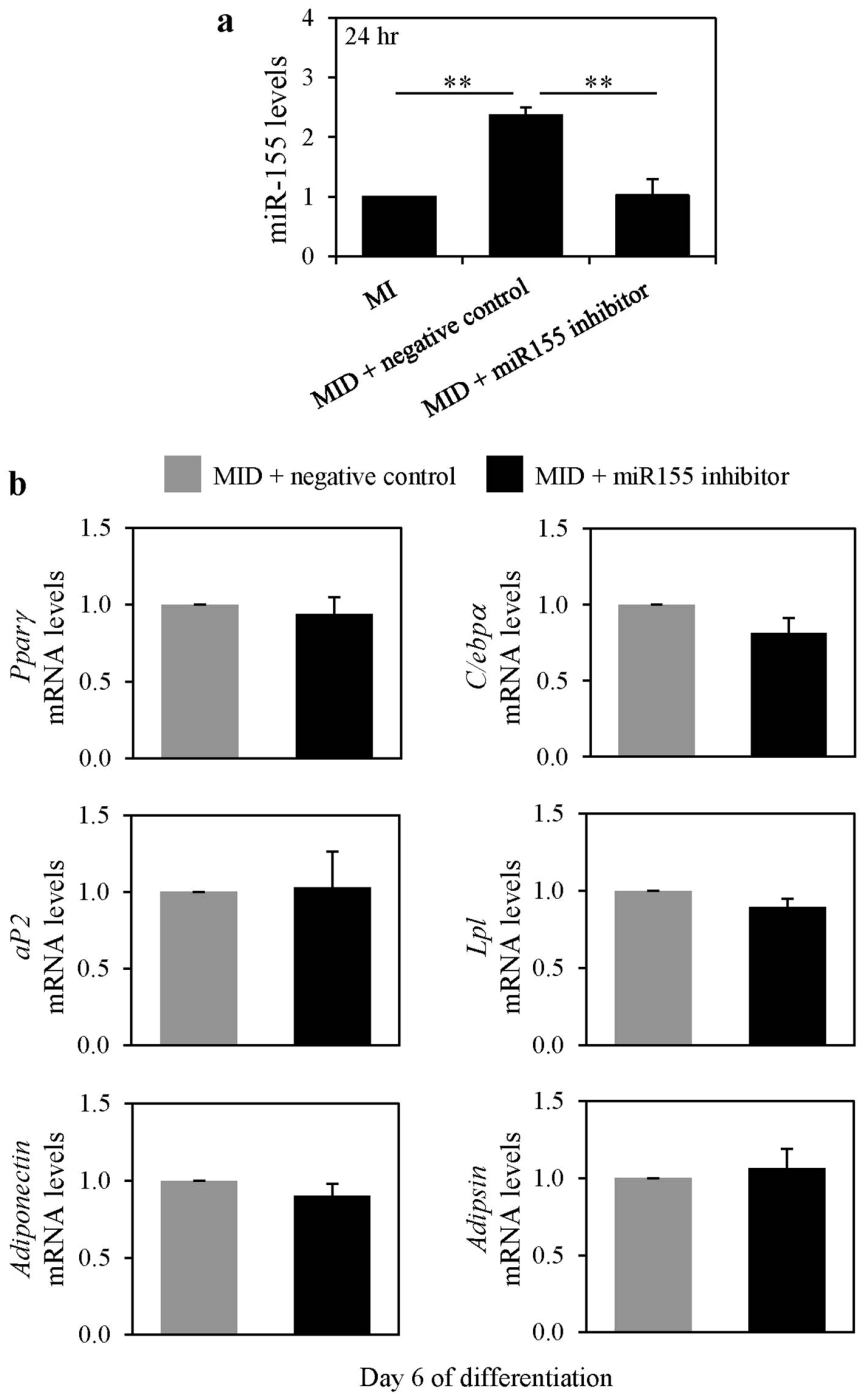
Dexamethasone mediated miR-155 up-regulation does not affect adipogenesis. Murine 3T3-L1 preadipocytes were transfected with miR-155 inhibitor or negative control. Cells were induced to differentiate in the presence of 500 µM IBMX (M), 100 nM insulin (I), and supplemented with either solvent control (MI) or 250 nM dexamethasone (MID) as indicated. (**a**) After 24 hours, miRNA was isolated, and the expression levels of miR-155 were quantified by real-time qPCR. Levels were normalized to endogenous RNU6 and expressed as fold over MI. Results from 3 separate experiments are graphically represented as mean ± S.E.M. (**b**) At day 6 of differentiation, RNA was isolated, and the mRNA levels of and adipogenic markers were quantified by real-time qPCR. Levels were normalized to endogenous *β-actin* levels and expressed as fold over MID + negative control. Results from 3 separate experiments are graphically represented as mean ± S.E.M. **Denotes p < 0.01 for the indicated comparisons, as assessed by one-way ANOVA with Tukey’s post-hoc tests.

To directly assess whether dexamethasone mediated endogenous miR-155 enhancement has a role in adipogenesis we induced 3T3-L1 preadipocytes to differentiate under MID conditions with either miR-155 inhibitor or negative control. After 24 hours the levels of miR-155 were measured by real time-qPCR in these cultures to validate that miR-155 enhancement in MID conditions was inhibited. Indeed, we show that after 24 hours miR-155 levels were increased under MID conditions (2.4-fold compared to basal MI levels) and this enhancement was abolished in the MID with the miR-155 inhibitor condition (Fig. 4a). At day 6 of differentiation, we assessed the mRNA expression levels of adipogenic markers by real time-qPCR in the cells differentiated with MID in the presence or absence of the miR-155 inhibitor. We found that inhibition of dexamethasone-mediated endogenous miR-155 enhancement did not affect the mRNA expression of adipogenic markers in differentiated 3T3-L1 adipocytes (Fig. 4b).

### Dexamethasone decreases the mRNA expression of miR-155 putative targets in differentiating preadipocytes

Since we established that dexamethasone-mediated miR-155 induction at the onset of differentiation might not be involved in adipogenesis, we next asked whether this miR-155 enhancement may have other roles. We used TargetScan prediction program (www.targetscan.org) to generate a list of potential candidate genes that have miR-155 target sites in the 3′-UTR of their mRNA. We also generated a list of genes that were suppressed by dexamethasone in a microarray analysis previously conducted in our laboratory (data not shown). We then compared the two lists and generated a short list of 9 candidate genes that were both putative targets of miR-155 and were found to be decreased with dexamethasone treatment in the microarray analysis.

**Figure 5:**
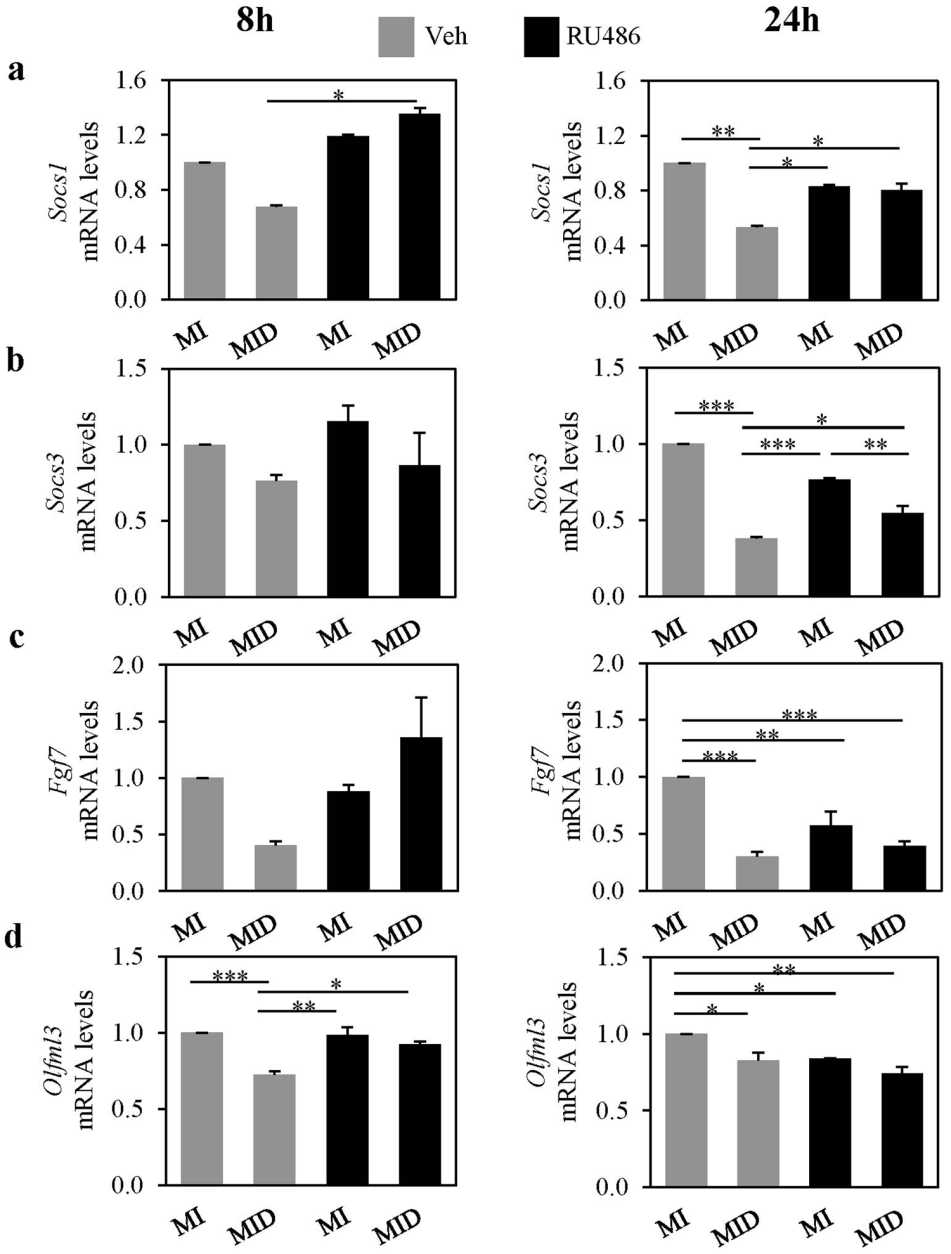
Dexamethasone treatment suppressed the expression of miR-155 putative targets in differentiating preadipocytes. Murine 3T3-L1 preadipocytes were induced to differentiate in the presence of 500 µM IBMX (M), 100 nM insulin (I), and supplemented with either solvent control (MI) or 250 nM dexamethasone (MID). Glucocorticoid receptor inhibitor RU486 (1 µM) or vehicle (Veh; ethanol) were added to MI and MID conditions as indicated. After 8 and 24 hours, RNA was isolated, and the expression levels of *Socs1* (**a**), *Socs3* (**b**), *Fgf7* (**c**) and *Olfml3* (**d**) were quantified by real-time qPCR. Levels were normalized to endogenous *β-actin* levels and expressed as fold over the MI + Veh control for each time point. Results from 3 separate experiments are graphically represented as mean ± S.E.M. *Denotes p < 0.05, **denotes p < 0.01, and ***denotes p < 0.001 for indicated pairs as assessed by one-way ANOVA with Tukey’s post-hoc tests.

To verify that these genes are decreased by dexamethasone and that they are indeed downstream of GR in differentiating preadipocytes, 3T3-L1 preadipocytes were induced to differentiate in MI versus MID conditions with or without the GR inhibitor, RU486. We measured the mRNA expression levels of candidate genes by real time-qPCR at 8 and 24 hours post treatment in these cultures. The mRNA levels of *suppressor of cytokine signaling* (*Socs*) 1 trended to decrease (32% reduction) in dexamethasone treated cells at 8 hours, and by 24 hours it was significantly reduced by 47% (Fig. 5a). The dexamethasone suppression of *Socs1* mRNA was normalized by RU486 at both time points, suggesting that *Socs1* is downstream of activated GR. The mRNA expression of *Socs3* followed a similar pattern, where it was trended to decrease (24%) at 8 hours, and by 24 hours it significantly decreased by 62% (Fig. 5b). Also, this dexamethasone mediated *Socs3* reduction was partially normalized by the GR inhibitor, RU486 at 24 hours, suggesting again, that *Socs3* is downstream of GR (Fig. 5b). Even though *Socs4* and *Socs6* were also potential candidates, they were not affected by dexamethasone treatment (Supplementary Fig. S2c and d). Dexamethasone also reduced the mRNA levels of *fibroblast growth factor 7* (*Fgf7*) by 59 and 70% at 8 and 24 hours respectively (Fig. 5c), and the reduction at 8 hours was normalized by RU486. The mRNA expression of *olfactomedin-like 3* (*Olfml3*) was also decreased by dexamethasone at 8 hours by 27% and this suppression was rescued by RU486 (Fig. 5d). The mRNA expression of other miR-155 putative targets such as *fos-like antigen 2* (*Fosl2*), *Cd47*, and *epithelial membrane protein 2* (*EMP2*) were all down regulated by dexamethasone treatment (Supplementary Fig. S2a,b and e). However, dexamethasone mediated down regulation of these genes was not normalized by RU486 after 24 hours. It should be noted that RU486 itself exerted some inhibitory effects which were evident in MI conditions- by 24-hour post treatment, where the mRNA levels of all genes tested were reduced (Fig. 5).

### Dexamethasone mediated suppressions of *Socs1*, *Socs3*, *Fgf7*, and *Olfml3* mRNA expressions are not via endogenous miR-155

**Figure 6:**
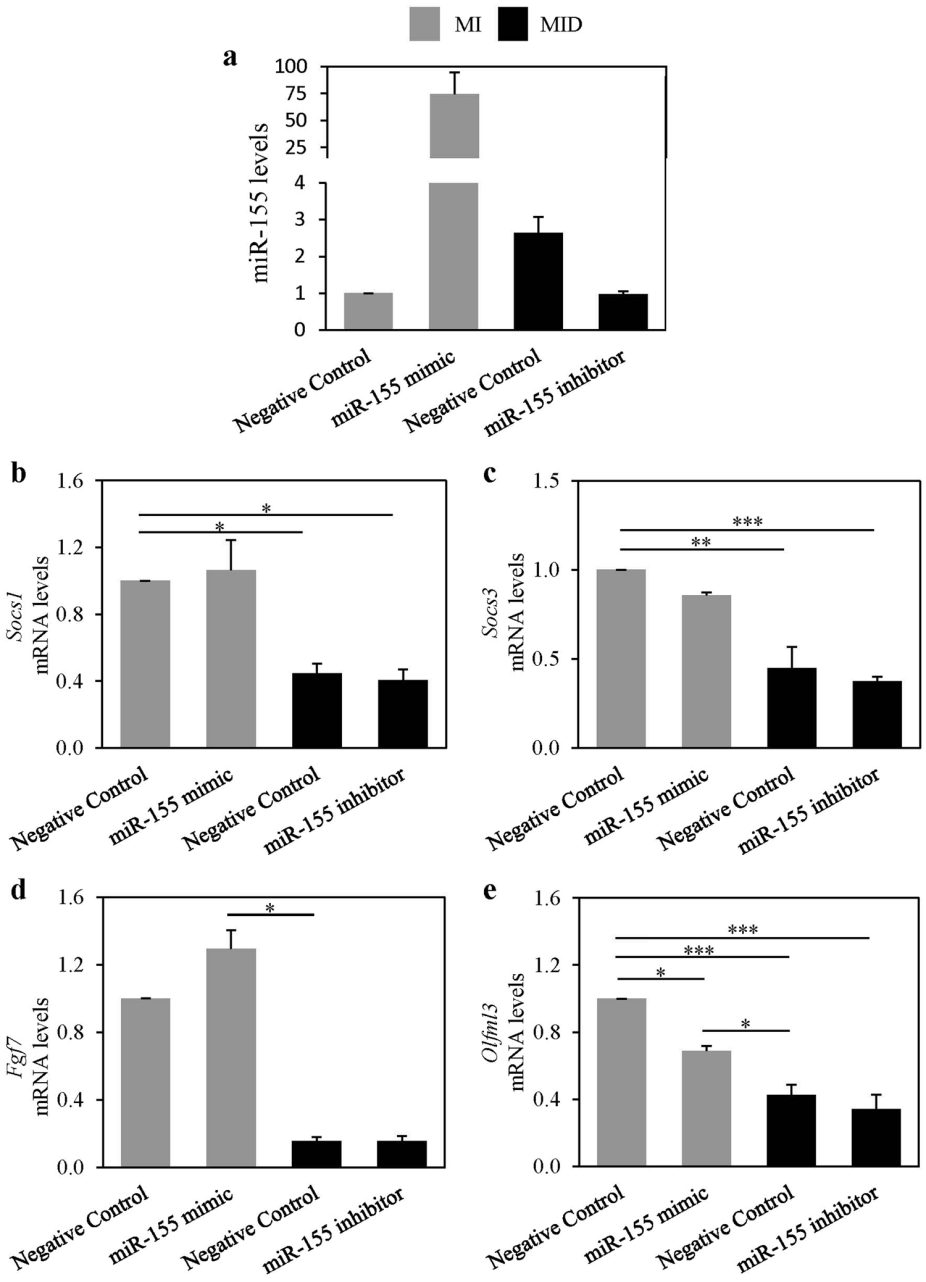
Dexamethasone induced mRNA reductions of *Socs1*, *Socs3*, *Fgf7*, and *Olfml3* are not mediated by miR-155 in differentiating preadipocytes. Murine 3T3-L1 preadipocytes were transfected with miR-155 mimic, miR-155 inhibitor, or negative controls. Cells were induced to differentiate in the presence of 500 µM IBMX (M), 100 nM insulin (I), and supplemented with either solvent control (MI) or 250 nM dexamethasone (MID) as indicated. After 48 hours, miRNA was isolated, and the expression levels of miR-155 (**a**), *Socs1* (**b**), *Socs3* (**c**), *Fgf7* (**d**) and *Olfml3* (**e**) were quantified by real-time qPCR. Levels were normalized to endogenous RNU6 (**a**) and *β-actin* (**b**–**e**) levels, and expressed as fold over the MI Negative Control. Results from 3 separate experiments are graphically represented as mean ± S.E.M. *Denotes p < 0.05, **denotes p < 0.01, and ***denotes p < 0.001 as assessed by one-way ANOVA with Tukey’s post-hoc tests.

Since dexamethasone treatment led to a robust decrease in the mRNA expressions of *Socs1*, *Socs3*, *Fgf7*, and *Olfml3* in differentiating preadipocytes, and we showed that this reduction was inhibited by the GR antagonist RU486, we asked whether this reduction occurred via miR-155. To examine this possibility, 3T3-L1 preadipocytes were transfected with miR-155 mimic, miR-155 inhibitor, or respective negative controls. Cells overexpressing miR-155 were treated with MI, whereas cells transfected with miR-155 inhibitor were treated with MID. The levels of miR-155 were validated by real time-qPCR in all cultures, and as expected the levels of miR-155 in the MI plus miR-155 mimic treatment were elevated (74-fold) compared to MI control (Fig. 6a). Also, as expected the levels of miR-155 in MID conditions increased by 2.7-fold compared to MI, and this increase was abolished almost entirely by the miR-155 inhibitor (Fig. 6a). We next measured the mRNA levels of *Socs1*, *Socs3*, *Fgf7*, and *Olfml3* in these cultures. We found that even after 48 hours dexamethasone suppression of the four genes was maintained. However, miR-155 overexpression did not mirror the effects of dexamethasone, except for *Olfml3*, which was reduced when the cells overexpressed miR-155. The miR-155 inhibitor did not restore the reduced mRNA expression of these genes (Fig. 6b–e). As such, these results indicate that *Socs1*, *Socs3*, and *Fgf7* mRNA are not targets of miR-155 in differentiating preadipocytes, and further, that miR-155 does not mediate the dexamethasone reduction of all four genes. In addition, the results show that *Olfml3* is a target of both miR-155 and dexamethasone; however, these responses are independent of each other.

## Discussion

To the best of our knowledge, we are the first to show that glucocorticoids increase the expression of endogenous miR-155 in 3T3-L1 preadipocytes and that this induction is dependent on GR transactivation. MiR-155 has been previously reported to be upregulated in a variety of cell types under inflammatory conditions^13,14,18,19^. The finding that miR-155 was upregulated by dexamethasone is particularly interesting since synthetic glucocorticoids are typically used in the clinic as anti-inflammatory agents^20^, and therefore one may anticipate that they would downregulate miR-155 expression. As previously mentioned, miR-155 expression is widely associated with inflammation in various cell types and studies assessing the role of glucocorticoids on miR-155 levels have done so primarily in the context of inflammation. For example, glucocorticoids have been reported to suppress the expression of miR-155 in activated and pro-inflammatory immune cells such as macrophages and T-cells^21,22^. Glucocorticoids also were shown to suppress miR-155 expression in livers of a lipopolysaccharides (LPS)-induced sepsis mouse model in a dose dependent manner^23^. However, the effects of glucocorticoids on naïve cells, which were not exposed to an inflammatory environment, were not assessed in these studies. Taken together these data and our findings in the present study suggest that glucocorticoid-mediated modulations of miR-155 levels may not only be cell-type dependent but also dependent on the presence of inflammatory factors in the local microenviroment of the cell.

The glucocorticoid mediated miR-155 increase we show in this study was detected in differentiating murine 3T3-L1 preadipocytes. Others have shown induction of miR-155 expression in murine 3T3-L1 preadipocytes and adipocytes but only when cells were exposed to the proinflammatory cytokine, TNF-α^13,14^. One study reported that under normal 3T3-L1 differentiation protocol, using insulin, IBMX, and dexamethasone, miR-155 levels were not altered as compared to preadipocytes; and that only TNFα- could induce miR-155 expression in preadipocytes^13^. These results are contradictory to our findings since we show that both dexamethasone (Fig. 1a) and IBMX (Fig. 1c) increased miR-155 levels compared to MI and preadipocytes respectively. Moreover, our GR inhibition studies demonstrated that that miR-155 is indeed a transcriptional target of GR (Fig. 2b).

We also found that with a PPARγ agonist (rosiglitazone) treatment the expression of miR-155 was reduced in differentiating preadipocytes (Fig. 1b). To the best of our knowledge, the effects of PPARγ agonists on miR-155 levels in differentiating preadipocytes have not been previously reported. Using insulin, IBMX, dexamethasone and a PPARγ agonist, one study reported that levels of miR-155 were decreased during differentiation of the human bone marrow-derived stromal cell line, hMSC-Tert20^15^. Although the effect of each differentiation inducer on miR-155 levels was not separately assessed, it may be plausible that the addition of a PPARγ agonist in the differentiation cocktail used for the hMSC-Tert20 cells could suppress a potential glucocorticoid mediated increase in miR-155. Here we show that dexamethasone but not rosiglitazone increased the expression of miR-155 in the 3T3-L1 cells. Therefore, it is plausible that rosiglitazone may be able to supress the dexamethasone-mediated miR-155 upregulation. Further, using the TRANSFAC® 7.0 software we identified two glucocorticoid responsive elements in the 5Kb sequence up-stream of miR-155. However, we could not find any PPAR responsive elements in this region indicating that PPAR is unlikely to directly bind to this sequence. Nevertheless, PPARγ may supress the expression of miR-155 indirectly through transrepression as has been shown for example for nuclear factor-κB^24^.

Our results show that the dexamethasone mediated increase in endogenous miR-155 did not affect adipogenesis. A well-cited paper reported that TNF-α mediated endogenous miR-155 induction in 3T3-L1 preadipocytes led to inhibition of adipogenesis. In their study TNF-α increased the levels of endogenous miR-155 in differentiating preadipocytes to similar levels as dexamethasone did in our study. However, since Liu *et al*. assessed adipogenesis only qualitatively by visualization of lipids and not quantitatively as we did in our study, one cannot directly compare our findings to theirs. Nonetheless, Liu *et al*. and others have shown that *ectopic* expression of miR-155 inhibits gene expression of adipogenic markers in differentiating preadipocytes and mature adipocytes^13–15^. In this study we show no effects of endogenous miR-155 upregulation on mRNA expression of adipogenic markers, however effects on protein levels cannot be ruled out.

Since endogenous glucocorticoid-induced miR-155 appeared to have no role in adipogenesis, we assessed other possible responses that could be mediated by glucocorticoids via miR-155. We show for the first time that dexamethasone treatment suppressed the mRNA expression of *Socs1* and *Socs3* in differentiating preadipocytes; and that the downregulation was dependent on GR activation since RU486 co-treatment abolished the effect of dexamethasone. SOCS family members are negative regulators of cytokine signalling and thus can modulate inflammatory responses^25^. In obesity, as a consequence of increased inflammatory cytokines, the levels of SOCS family members increase in the insulin sensitive peripheral tissues including white adipose tissue, muscle, and liver^26^. Upregulation of SOCS1 and SOCS3 leads to inhibition of insulin signalling in various cell types- including adipocytes^27^. The role(s) of SOCS proteins in adipogenesis and/or adipocyte biology are not very well understood. Under high-fat diet, both SOCS1 (global knockout) and SOCS3 (liver specific knockout) deficiencies in mice have been associated with increased visceral (epididymal) fat accumulation^28,29^. Chronic exposure to glucocorticoids leads to the development of visceral obesity^1^, however, it is not known whether this glucocorticoid associated visceral obesity could be mediated by decreased SOCS1 or SOCS3 expression in differentiating preadipocytes.

*Socs1* and *Socs3* are known targets of miR-155 in various cell-types^30–34^. However, we showed that dexamethasone mediated decrease in *Socs1* and *Socs3* mRNA in differentiating preadipocytes was not via endogenous miR-155. Interestingly, overexpression of miR-155 also did not suppress the expression of *Socs1* and *Socs3* in these cells. In other cell types, such as murine microglia and human macrophages, overexpression of miR-155 suppressed the expression of *Socs1* and these responses were normalized when miR-155 was inhibited^32,33^. Similarly, miR-155 reduced *Socs3* levels in human oligodendrocytes^34^. Taken together, these data suggest that the role of miR-155 in modulating *Socs1* and *Socs3* expression may be species and cell type specific.

We also showed that dexamethasone treatment suppressed *Fgf7* and *Olfml3* mRNA expression in differentiating preadipocytes and that these reductions were dependent on GR. Others have demonstrated that dexamethasone treatment of murine fibroblasts suppressed the mRNA expression of *Fgf7* in a time and dose dependent manner^35^. FGF7 is a growth factor that is involved in preadipocyte proliferation and differentiation^36^. Since 3T3-L1 preadipocytes must reach cellular senescence before they can start differentiating, it is plausible that *Fgf7* expression would decrease with differentiation since it would no longer be required^37^. Furthermore, we found that the dexamethasone mediated reduction of *Fgf7* mRNA was not via miR-155 in differentiating 3T3-L1 preadipocytes. *Fgf7* mRNA is reported to be a target of miR-155 as assessed by luciferase assays in murine and human lung fibroblasts systems^38^. However, in the present study although *Fgf7* was inhibited by dexamethasone, it was not inhibited by miR-155. These data suggest once more that the role of miR-155 in modulating gene expression may be distinct in 3T3-L1 preadipocytes compared to other cell types.

OLFML3 is a secreted scaffold protein involved in facilitating cell adhesion, protein-protein, and intercellular interactions^39^. In human macrophages, glucocorticoids modestly suppressed the gene expression of *Olfml*3, which is in accordance with our data in this study^40^. The mRNA expression of *Olfml3* has been shown to be decreased by miR-155 in a porcine kidney epithelial cell line^41^. In this study we also showed in differentiating preadipocytes that overexpression of miR-155 reduced the mRNA levels of *Olfml3*. However, we further showed that the observed dexamethasone mediated reduction of *Olfml3* mRNA levels was not via endogenous miR-155. Taken together these results once more may point to a differential role for endogenous versus exogenous sources of miR-155 in differentiating preadipocytes, and that GR may supress the expression of these genes independently of miR-155.

## Conclusion

In the present study we show that the synthetic glucocorticoid, dexamethasone, increased endogenous miR-155 levels in differentiating preadipocytes. We showed that this dexamethasone mediated miR-155 increase does not affect adipogenesis despite previous reports that ectopic overexpression of miR-155 has an inhibitory role in adipogenesis. These data and our findings indicate that the effects of miR-155 on differentiating preadipocytes may be dependent on the source of miR-155 and the critical dose. Perhaps as a secreted factor from other cell types in the adipose tissue, miR-155 would inhibit adipogenesis whereas endogenous miR-155 in differentiating preadipocytes may have other roles, which are still to be discovered.

## Methods

### Cell culture and preadipocyte differentiation

3T3-L1 mouse embryonic fibroblasts were maintained in DMEM containing 5.6 mM glucose (Hyclone, Mississauga, ON, Canada), supplemented with 10% bovine calf serum (ATCC, Manassas, VA, USA) and 1% penicillin/streptomycin (P/S; Life Technologies, Burlington, ON, Canada). Once at 70% confluence, cells were plated in 6-well dishes and left to reach confluence. Two days post-confluence (day 0) cells were induced to differentiate in DMEM containing 5.6 mM glucose supplemented with 10% fetal calf serum (Wisent, Montreal, QC, Canada) and 1% P/S (Life Technologies, Burlington, ON, Canada). Differentiation was induced using 500 µM 3-isobutyl-1-methylxanthine (IBMX) and 100 nM insulin (Roche Diagnostics, Laval, QC, Canada) (MI), with either 250 nM dexamethasone (MID), 5 µM rosiglitazone (MIR), or solvent (ethanol) control (MI). At day 2 and 4 of differentiation, media was replaced to contain 100 nM insulin. For GR inhibitor studies, RU486 (1 µM) was added to MID conditions at the start of preadipocyte differentiation (all Sigma-Aldrich, Oakville, ON, Canada unless otherwise indicated) Cells were harvested at indicated times during differentiation process.

### Transfection analysis

For transfection analysis, 3T3-L1 preadipocytes were maintained as described above and seeded in 6 well plates to reach 85–95% confluence. Cells were then transfected with miR-155 mimic (1.5 pmol), miR-155 inhibitor (15 pmol), or negative controls using Lipofectamine reagent (all from Life Technologies, Burlington, ON, Canada) following the manufacturer’s instructions. Following transfection, cells were left to reach confluence and then treated for differentiation as described above.

### RNA extraction and real-time quantitative PCR (real time-qPCR)

Total RNA was extracted from differentiating preadipocytes treated as previously described. For target mRNA detection, RNA was isolated at indicated time points using the RNeasy Mini kit where genomic DNA was removed using the RNase-Free DNase Kit (Qiagen, Mississauga, ON, Canada). RNA (0.5 µg) was reverse transcribed using iScript cDNA Synthesis Kit (Bio-Rad, Mississauga, ON, Canada) following the manufacturer’s recommendations. Sample cDNA was amplified and quantified in a CFX96-PCR Detection System using the iQSYBR SsoFast EvaGreen Supermix (Bio-Rad, Mississauga, ON, Canada). The primer pairs for each gene target are summarized in Supplementary Table S2. All genes were normalized to *β-actin* levels and analysed using the comparative CT (ΔΔCT) method.

For target miRNA detection RNA was isolated using *mir*Vana miRNA isolation kit (Life Technologies, Burlington, ON, Canada) following the manufacturer’s instructions. RNA (0.5 µg) was reverse transcribed using miScript II RT Kit (Qiagen, Mississauga, ON, Canada) following the manufacturer’s recommendations. Sample cDNA was amplified and quantified in a CFX96-PCR Detection System using the miScript SYBR Green PCR Kit with miScript Primer Assays for miR-155 and RNU6 (Qiagen, Mississauga, ON, Canada). Levels of miR-155 were normalized to RNU6 and data were analysed using the comparative CT (ΔΔCT) method.

### Lipid staining and quantification

Murine 3T3-L1 preadipocytes were differentiated as described above. At day 8 of differentiation cells were fixed using paraformaldehyde (4%; Electron Microscopy Sciences, Hatfield, PA, USA) and stained with Nile Red (stains cytoplasmic lipid droplets; Sigma Aldrich) and DAPI (stains cell nuclei; Sigma Aldrich, Oakville, ON, Canada) as previously described^42^. Nile Red fluorescence was quantified at 485/528 nm (excitation/emission) and normalized to DAPI staining measured at 360/460 (excitation/emission). All data were then normalized to MI control (data reported as fold-change over MI). Fluorescence was measured using the Synergy 2 Microplate Reader (BioTek Instruments Inc., Winooski, VT, USA). Images of Nile Red and DAPI staining were taken using the Leica TCD SP8 confocal microscope (Leica Microsystems, Toronto, ON, Canada) at 10× magnification.

### Statistical Analyses

One-way ANOVA, followed by Tukey’s post-hoc tests were used when comparing multiple means within an experiment. Paired t-tests were used when comparing two means. Significance was defined as p < 0.05. Statistical analyses were performed using SigmaPlot software 12.5 (San-Jose, CA, U.S.A.). Shapiro-Wilk test was used to ascertain normality of the data.

### Data Availability

The datasets generated during and/or analysed during the current study are available from the corresponding author on reasonable request.

## Electronic supplementary material

Supplementary information
