# Supplementary material for: Dexamethasone induced miR-155 up-regulation in differentiating 3T3-L1 preadipocytes does not affect adipogenesis
Source: Sci Rep. 2018 Jan 19;8:1264. doi: 10.1038/s41598-018-19704-4 (PMC5775309; doi:10.1038/s41598-018-19704-4)
Supplement: Supplementary file 1 — Supplementary information [file 41598_2018_19704_MOESM1_ESM.pdf]

Supplementary Information

Dexamethasone induced miR-155 up-regulation in differentiating 3T3-L1 preadipocytes does not affect adipogenesis.

Vian Peshdary and Ella Atlas

miRNA	Fold Change	FDR p-value
mmu-miR-29b-3p	1.59	0.005
mmu-miR-20a-5p	1.52	0.000
mmu-miR-92a-3p	1.42	0.033
mmu-miR-20b-5p	1.34	0.010
mmu-miR-19b-3p	1.32	0.030
mmu-miR-155-5p	1.30	0.000
mmu-miR-221-3p	1.29	0.005
mmu-miR-146b-5p	1.18	0.048
mmu-miR-21-3p	1.10	0.000
mmu-miR-883a-5p	1.06	0.030
mmu-miR-3971	-1.04	0.005
mmu-miR-344d-3p	-1.04	0.048
mmu-miR-3080-5p	-1.05	0.010
mmu-miR-3112-3p	-1.08	0.010
mmu-miR-351-3p	-1.10	0.026
mmu-miR-2861	-1.27	0.010
mmu-miR-125b-5p	-1.40	0.019
mmu-miR-503-5p	-1.50	0.000

Supplementary Table S1. MiRNA array analysis evaluating miRNA profiles in differentiating 3T3-L1 preadipocytes treated with dexamethasone. Murine 3T3-L1 preadipocytes were induced to differentiate in MI (IBMX and insulin) compared to MID (IBMX, insulin, and dexamethasone) conditions. After 24 hours, miRNA was collected and used for miRNA array analysis. The miRNAs with significant fold changes (false discovery rate (FDR) p-value <0.05) comparing MID to MI are shown.

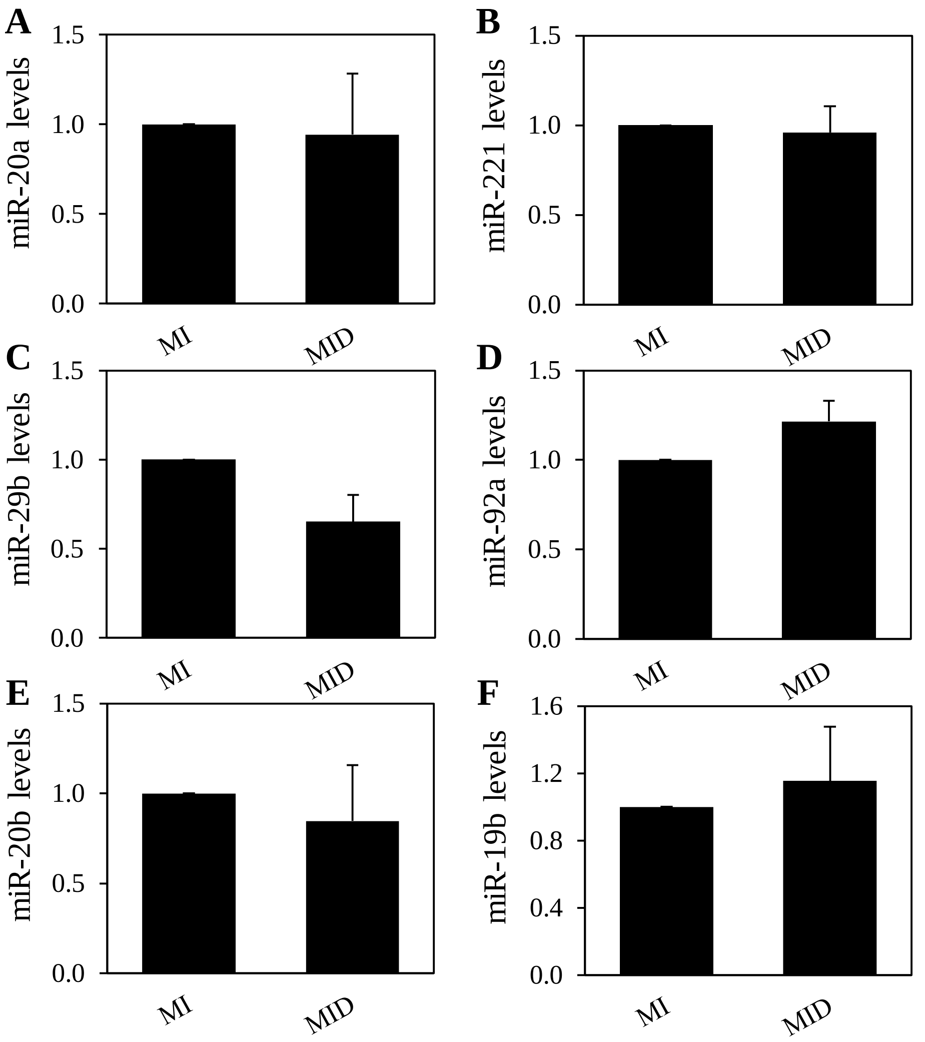

Supplementary Figure S1. **MiRNA array validation of changed miRNAs in response to dexamethasone**. Murine 3T3-L1 preadipocytes were induced to differentiate in the presence of 500 µM IBMX (M), 100 nM insulin (I) and supplemented with either solvent control (MI) or 250 nM dexamethasone (MID). After 24 hours, miRNA was isolated, and the expression levels of indicated miRNAs were quantified by real-time qPCR. Levels were normalized to endogenous RNU6 and expressed as fold over MI. Results from 3 separate experiments are graphically represented as mean ± S.E.M.

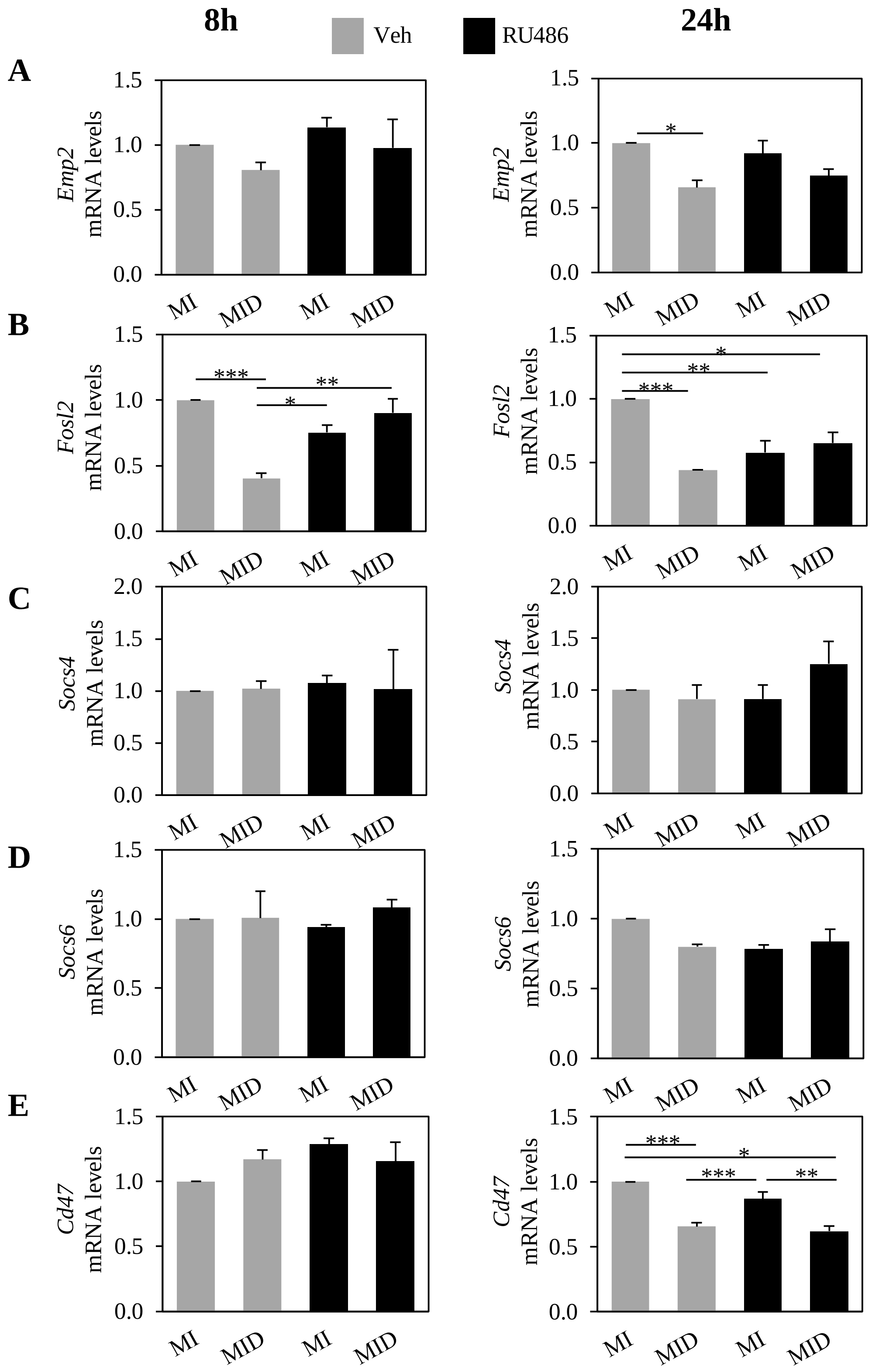

Supplementary Figure S2. Effect of dexamethasone treatment on the expression of miR-155 putative targets in differentiating preadipocytes. Murine 3T3-L1 preadipocytes were induced to differentiate in the presence of 500 µM IBMX (M), 100 nM insulin (I), and supplemented with either solvent control (MI) or 250 nM dexamethasone (MID). Glucocorticoid receptor inhibitor RU486 (1 µM) or vehicle (Veh) were added to MI and MID conditions as indicated. After 8 and 24 hours, RNA was isolated, and the expression levels of *Emp2* (**A**), *Fosl2* (**B**), *Socs4* (**C**), *Socs6* (**D**), and *Cd47* (**E**) were quantified by real-time qPCR. Levels were normalized to endogenous *β-actin* levels and expressed as fold over the MI+Veh control for each time point. Results from 3 separate experiments are graphically represented as mean ± S.E.M. * denotes $p<0.05$, ** denotes $p<0.01$, and *** denotes $p<0.001$ for indicated pairs as assessed by one-way ANOVA with Tukey's post-hoc tests.

Target	Primer	Sequence
Pparγ	Forward	5'-GCCTGCGGAAGCCCTTTGGT-3'
	Reverse	5'-GCAGTTCCAGGGCCTGCAGC-3'
C/ebpα	Forward	5'-TGCGCAAGAGCCGAGATAAA-3'
	Reverse	5'-CCTTGACCAAGGAGCTCTCA-3'
aP2	Forward	5'-GGAAGCTTGTCTCCAGTGAA-3'
	Reverse	5'-GCGGTGATTTCATCGAATTC-3'
Lpl	Forward	5'-CAGGATGTGGCCCGGTTTAT-3'
	Reverse	5'-CGGGGCTTCTGCATACTCAA-3'
Adiponectin	Forward	5'- TGACGACACCAAAAGGGCTC -3'
	Reverse	5'- CACAAGTTCCCTTGGGTGGA -3'
Adipsin	Forward	5'- CCTGAACCCTACAAGCGATG -3'
	Reverse	5'- CAACGAGGCATTCTGGGATA -3'
Socs1	Forward	5'-TGGTAGCACGCAACCAGGTG-3'
	Reverse	5'-TGGCGAGGACGAAGACGAG-3'
Socs3	Forward	5'-TAGACTTCACGGCTGCCAAC-3'
	Reverse	5'-CGGGGAGCTAGTCCCGAA-3'
Socs4	Forward	5'-CGGAGAGATCCGTCCAGAAA-3'
	Reverse	5'-GCACTTCGACTCCGACTTGT-3'
Socs6	Forward	5'-GCGGCTGCAGGGTTTTCATT-3'
	Reverse	5'-TGCCTCATGGGTGTATTCTGC-3'
Fgf7	Forward	5'-ACGGCTACGAGTGTGAACTG-3'
	Reverse	5'-CCATGATGTTGTAGCTGTTCTTCA-3'
Emp2	Forward	5'-GTGCCCGGTTTGGATTTGTC-3'
	Reverse	5'-CGTGGACACGATGTGGAAGA-3'
Fosl2	Forward	5'-TATCCCGGGAACTTTGACACC-3'
	Reverse	5'-ATATCTACCCGGAACTTCTGCTG-3'
Olfml3	Forward	5'-ACGATATGGTGACGGACTGT-3'
	Reverse	5'-AGGCCAACTGAACCACCAAA-3'
Cd47	Forward	5'-CGATGCCATGGTGGGAAACT-3'
	Reverse	5'-TCAGTGTTGAAGGCCGTGC-3'
β-actin	Forward	5'- GACTTCGAGCAAGAGATGGC -3'
	Reverse	5'- CCAGACAGCACTGTGTTGGC -3'

Supplementary Table S2. Primer sequences used for real-time qPCR analysis.
